# Harnessing Placebo Effects in Primary Care: Using the Person-Based Approach to Develop an Online Intervention to Enhance Practitioners' Communication of Clinical Empathy and Realistic Optimism During Consultations

**DOI:** 10.3389/fpain.2021.721222

**Published:** 2021-08-24

**Authors:** Kirsten A. Smith, Jane Vennik, Leanne Morrison, Stephanie Hughes, Mary Steele, Riya Tiwari, Jennifer Bostock, Jeremy Howick, Christian Mallen, Paul Little, Mohana Ratnapalan, Emily Lyness, Pranati Misurya, Geraldine M. Leydon, Hajira Dambha-Miller, Hazel A. Everitt, Felicity L. Bishop

**Affiliations:** ^1^Primary Care Population Sciences and Medical Education, University of Southampton, Southampton, United Kingdom; ^2^Centre for Clinical and Community Applications of Health Psychology, Department of Psychology, University of Southampton, Southampton, United Kingdom; ^3^Policy Innovation & Evaluation Research Unit, London School of Hygiene & Tropical Medicine, London, United Kingdom; ^4^Faculty of Philosophy, University of Oxford, Oxford, United Kingdom; ^5^Primary Care Centre Versus Arthritis, School of Medicine, Keele University, Keele, United Kingdom

**Keywords:** placebo effects, primary medical care, doctor patient communication, clinical empathy, optimism, osteoarthritis, qualitative research, pain

## Abstract

**Background:** Empathic communication and positive messages are important components of “placebo” effects and can improve patient outcomes, including pain. Communicating empathy and optimism to patients within consultations may also enhance the effects of verum, i.e., non-placebo, treatments. This is particularly relevant for osteoarthritis, which is common, costly and difficult to manage. Digital interventions can be effective tools for changing practitioner behavior. This paper describes the systematic planning, development and optimization of an online intervention—“Empathico”–to help primary healthcare practitioners enhance their communication of clinical empathy and realistic optimism during consultations.

**Methods:** The Person-Based Approach to intervention development was used. This entailed integrating insights from placebo and behavior change theory and evidence, and conducting primary and secondary qualitative research. Systematic literature reviews identified barriers, facilitators, and promising methods for enhancing clinical empathy and realistic optimism. Qualitative studies explored practitioners' and patients' perspectives, initially on the communication of clinical empathy and realistic optimism and subsequently on different iterations of the Empathico intervention. Insights from the literature reviews, qualitative studies and public contributor input were integrated into a logic model, behavioral analysis and principles that guided intervention development and optimization.

**Results:** The Empathico intervention comprises 7 sections: Introduction, Empathy, Optimism, Application of Empathico for Osteoarthritis, Reflection on my Consultations, Setting Goals and Further Resources. Iterative refinement of Empathico, using feedback from patients and practitioners, resulted in highly positive feedback and helped to (1) contextualize evidence-based recommendations from placebo studies within the complexities of primary healthcare consultations and (2) ensure the intervention addressed practitioners' and patients' concerns and priorities.

**Conclusions:** We have developed an evidence-based, theoretically-grounded intervention that should enable practitioners to better harness placebo effects of communication in consultations. The extensive use of qualitative research throughout the development and optimization process ensured that Empathico is highly acceptable and meaningful to practitioners. This means that practitioners are more likely to engage with Empathico and make changes to enhance their communication of clinical empathy and realistic optimism in clinical practice. Empathico is now ready to be evaluated in a large-scale randomized trial to explore its impact on patient outcomes.

## Introduction

Placebo effects can be substantial and clinically meaningful; efforts to harness them in clinical practice to benefit patients are therefore warranted (1). There are at least two main ways in which this can be approached, depending on one's definition of placebo effects. Traditional, substance-based, definitions of placebo effects hold that placebo effects are elicited by the administration of a placebo substance (e.g., the archetypal “sugar pill”) (2). From this perspective, harnessing placebo effects in clinical practice requires the prescription of placebos; concerns over the ethics of deceptive prescribing in clinical settings have led researchers to examine the effects of prescribing open label placebos. A different approach is suggested by process-oriented definitions of placebo effects, in which placebo effects are elicited by the psychosocial context within which treatment occurs and, especially, the doctor-patient interaction (2). From this perspective, harnessing placebo effects in clinical practice can be achieved by leveraging the psychosocial context that triggers the neuropsychological processes underpinning placebo effects. This approach also aligns with data suggesting that clinicians and patients may be more favorably inclined toward harnessing placebo effects through leveraging psychosocial context than through prescribing placebos (2, 3). It is this process-oriented perspective that guided our intention to develop an intervention to enable primary care practitioners to harness placebo effects by enhancing their communication of clinical empathy and realistic optimism in clinical consultations.

While there are multiple processes that occur within the psychosocial context that might trigger the neuropsychological processes underpinning placebo effects (4) we chose to focus specifically on clinicians' communication of clinical empathy and realistic optimism. This decision was guided by an analysis of key behavioral considerations according to the Behavior Change Wheel, which provides a systematic, theory-driven, “top-down,” approach to specifying the behavior changes, components, and techniques likely to make interventions effective (5). The Behavior Change Wheel was developed based on a review of 19 existing frameworks and expert consultation, and was designed to be comprehensive, coherent, and to clearly link to an overarching model of behavior (5). Specifically, we considered the likely impact of the intended behavior change, the likelihood of being able to actually change each behavior, the likelihood of spill-over (to other individuals/settings) and the ease of measurement of each behavior.

We considered the likely impact of improving communication of clinical empathy and realistic optimism to be high. Clinical empathy involves the practitioner putting themselves in a patient's position, acknowledging their feelings, concerns and expectations and behaving in a way that communicates that understanding (6, 7). A compassionate, friendly consultation style using appropriate non-verbal cues can enhance the management of pain and related conditions and has been associated with greater patient satisfaction, adherence to treatment, and quality of life and health outcomes (8–10). Clinical empathy can also be beneficial to practitioners in reducing stress and burnout (11). Empathic communication has even been proposed as an essential prerequisite for enabling people to better cope with, understand, and self-manage their health (12).

Patients' positive expectancies about treatment outcomes are associated with better outcomes in laboratory and clinical studies of diverse symptoms, especially pain (13–15) and are an important part of the neuropsychological processes underpinning placebo effects (16, 17). For example, positive expectancies of analgesia alter pain perception via effects on central nervous system processing (18) and trigger a cascade of neurological changes that are very similar to those triggered by pharmaceutical analgesics (19). However, some of the methods used in placebo experiments to impart positive outcome expectancies, such as positive messages in the form of short verbal statements that an intervention is a potent painkiller, may not be convincing for patients with pain in clinical practice (20, 21). Furthermore, for healthcare practitioners “expectancies” and “expectations” are terms associated with “expectation management” which typically involves encouraging patients to have more realistic beliefs about the outcomes of treatment; for example, a patient may expect a hip replacement within a few months of experiencing moderate osteoarthritis (OA) pain but this is unlikely to be the most appropriate initial management strategy, “expectation management” in this context involves tailored education on OA pain explaining the potential benefits of other options such as exercise, weight loss and analgesia prior to considering surgery and a realistic assessment of the risks vs. the potential benefits of surgery. Our digital intervention aims to promote effective ways of encouraging patients to have positive outcome expectancies, within the context of their clinical situation—hence our focus on *realistic* optimism, within an empathic practitioner-patient interaction (22).

The extensive literature on communication skills training suggested that we would be able to change practitioners' communication of clinical empathy and realistic optimism. As has been discussed by others (23), placebo studies can be seen to overlap with studies on doctor-patient communication and relationships, as well as topics such as patient-centered care more broadly. Our intervention is a good example of this overlap, as our fundamental aim is to enhance primary care practitioners' communication with their patients. Practitioners are generally willing to engage in communication skills training and evidence shows this training can be successful at changing the target behaviors (24–26). However, there is insufficient evidence that shows that training a practitioner impacts upon a patient's health (27). Moreover, there has been little consensus on what communication skills training should entail with most interventions being complex, expensive and time-consuming (24).

Considering the broad relevance of good communication in clinical practice, we considered there to be good potential for wider impact to other individuals and settings. We initially chose to focus on enhancing practitioners' communication of clinical empathy and realistic optimism in consultations with patients with OA. OA is a common, costly, and painful condition (28, 29). It is a top 20 cause of disability adjusted life years globally (30) and it can significantly impair quality of life (31) and function (32). Research indicates there is scope to improve practitioner communication with patients with OA (33) and improving communication can significantly improve OA pain (34). Improving communication and person-centered care is an important goal in healthcare worldwide (35). Excellent practitioner-patient communication has been shown to significantly improve patients' adherence to treatment, quality of life and satisfaction, comparable to pharmaceutical interventions (7, 25, 36). Moreover, poor consultations can have negative impacts on patients, such as non-adherence to treatment, decreased quality of life, increased costs and increased complaints and litigation (7). Practitioners typically draw on the same repertoire of communication behaviors for all consultations, thus learning new communication behaviors within the context of one condition is likely to also enhance communication in consultations for other conditions. Improving patient-practitioner communication can therefore have wide-ranging benefits for patients and health services.

The work presented in this paper aimed to plan and optimize a definitive, replicable, testable, and implementable brief digital intervention (DI) – called Empathico – to enhance primary healthcare practitioners' communication of clinical empathy and realistic optimism in consultations with patients presenting with OA. By describing our approach, we illustrate one way in which it is possible to identify, specify, and address the challenges of translating findings from placebo studies into clinical practice in a way that ensures findings can and will be implemented by healthcare practitioners for the benefit of patients. The challenges we have identified and our approaches to addressing them may be of interest to others also wanting to harness placebo effects and improve associated communication skills in clinical practice.

## Methods Overview

### Ethical Approvals

Ethical approvals for all the studies in this paper were obtained from the National Research Ethics Service West Midlands-South Birmingham Research Ethics Committee (19/WM/0027 25th Jan 2019). All participants received a participant information sheet, were given the opportunity to ask questions and gave informed consent prior to taking part in the studies.

### Public and Patient Involvement

Four public contributors with OA have been involved in different ways in different parts of the project including: as a full member of the project management group that met monthly to monitor progress and make key design decisions; contributing to patient-facing documents and interview topic guides; reviewing study protocols and commenting on ethics applications; providing feedback on intervention content; assisting with the analysis and interpretation of results; and contributing to article writing.

### Design

**Figure 1:**
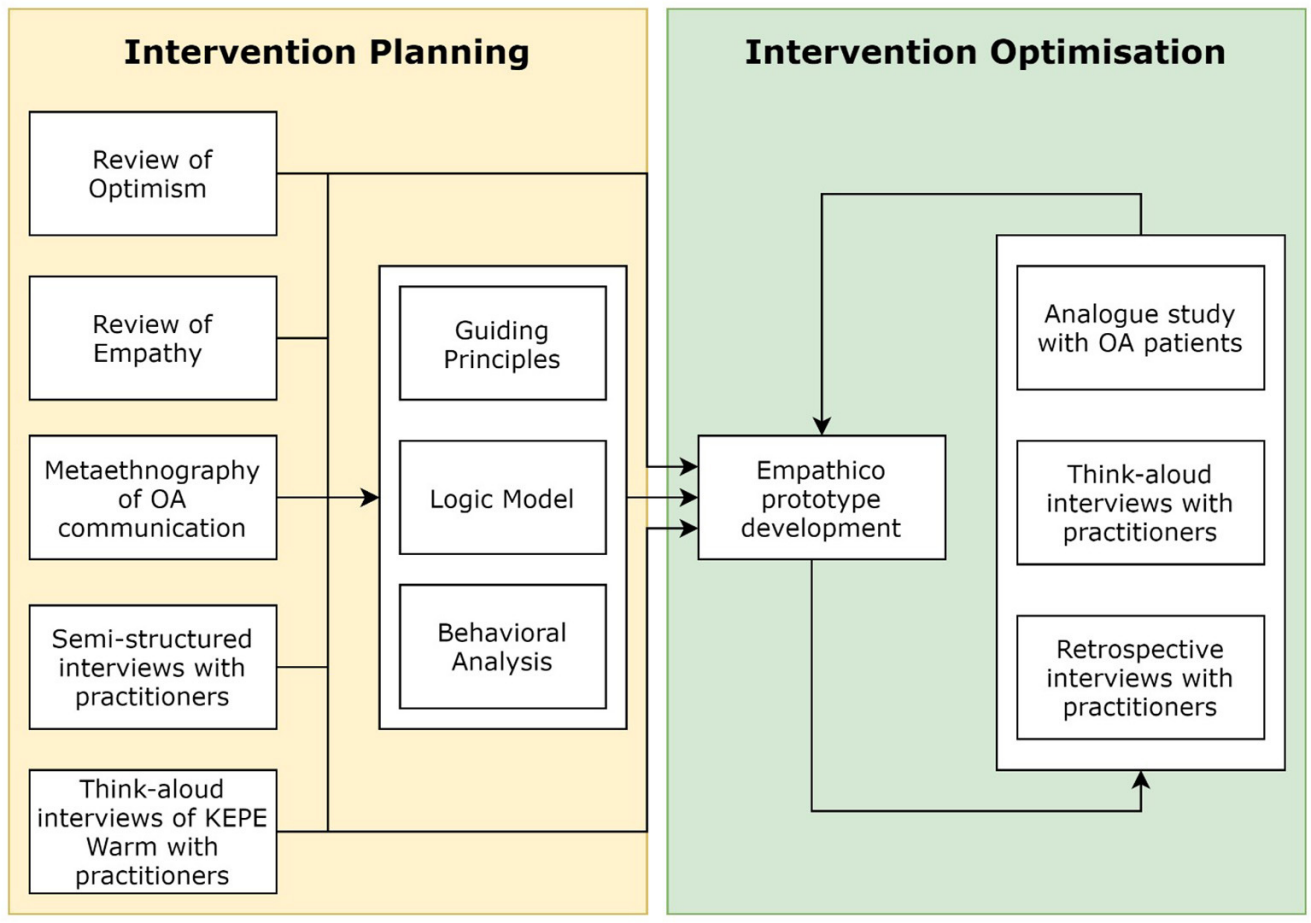
Overview of studies and activities conducted within the intervention planning and optimization phases of Empathico's development.

We used the Person-Based Approach (PBA) (37) to develop the digital intervention. The PBA involves extensive qualitative research which can be integrated alongside theory and evidence mapping to assess the problem area, develop and iteratively refine an intervention. Using the PBA increases the likelihood that target users will engage with an intervention and minimizes resource waste from trialing a suboptimal intervention. Interventions must be used and engaged with in a meaningful way to successfully mediate behavior change. The concept and process of “effective” engagement is dynamic and multifaceted; users need to sufficiently engage with both the physical intervention and target behaviors, which can occur at a behavioral (e.g., logging in, practicing target behaviors etc.) and experiential (e.g., interest, perceived utility, relevance, practicality etc.) level and can be shaped by a range of contextual factors such as social support and organizational culture (38, 39). This meant that multiple mixed method studies were needed to adequately understand and optimize practitioners' engagement with Empathico. The PBA process has two main phases, intervention planning and optimization. Figure 1 depicts the studies that we conducted as part of intervention planning and optimization and shows the outputs of each phase. Some of these studies have been or are being published separately as stand-alone papers where readers will find full methodological details; the current paper explicates how the findings from these studies were used to develop our intervention. Table 1 defines some of the technical terms associated with the PBA that we refer to throughout this paper.

**Table 1:** Glossary of technical terms associated with the PBA.

**Term**	**Definition**
Person-Based Approach (PBA)	A systematic approach to developing digital interventions that involves extensive (primary and/or secondary) qualitative research to focus on and elucidate intervention users' engagement with the intervention. The PBA is typically integrated alongside theory and evidence mapping to assess the problem area, develop and iteratively refine an intervention (37).
Guiding Principles	Design objectives that the intervention must address to be optimally meaningful, relevant, acceptable, and practical for users. Guiding Principles also specify design features that will address those objectives.
Logic Model	A visual representation that maps how the intervention is hypothesized to effect change in the intended outcomes. Specifies variables that are thought to operate along the causal pathway between exposure to the intervention and its ultimate effects on health outcomes.
Behavioral Analysis	An analysis of the behaviors that must occur if a recipient is to engage effectively with the intervention, to initiate and maintain the intended behaviors. Includes identification of determinants (facilitators and barriers) of behavior change and techniques that are likely to support the intended behavior change.

### Participants

In total, 39 primary healthcare practitioners and 33 patients with OA took part in our intervention development studies. Participants were recruited from primary care settings in Southern England and recruitment was supported by the Wessex NIHR Clinical Research Network. Table 2 summarizes the characteristics of participants overall and in each of the studies reported in this paper.

**Table 2:** Demographic characteristics of study participants.

**Phase:**	**Planning Phase**				**Optimization Phase**						**Overall**	
**Study:**	**Practitioner interviews (*****n*** **= 20)**		**KEPE-Warm think-aloud (*****n*** **= 7)**		**Patient interviews (*****n*** **= 33)**		**Empathico think-aloud (*****n*** **= 15)^a^**		**Practitioner retrospective (*****n*** **= 5)^b^**		**Total (*****n*** **= 39 practitioners, 33 patients)**	
	***n***	**%**	***n***	**%**	***n***	**%**	***n***	**%**	***n***	**%**	***n***	**%**
**Role**												
Physiotherapist	2	10%	0	0%	0	0%	0	0%	2	40%	3	4%
Nurse	2	10%	0	0%	0	0%	0	0%	2	40%	4	6%
GP	16	80%	3	43%	0	0%	15	100%	1	20%	28	39%
GP Trainee	0	0%	4	57%	0	0%	0	0%	0	0%	4	6%
Patient	0	0%	0	0%	33	100%	0	0%	0	0%	33	46%
**Ethnicity**												
White	18	90%	0	0%	33	100%	14	93%	5	100%	62	86%
Asian	1	5%	0	0%	0	0%	1	7%	0	0%	2	3%
Other	1	5%	0	0%	0	0%	0	0%	0	0%	1	1%
Unknown	0	0%	7	100%	0	0%	0	0%	0	0%	7	10%
**Gender**												
Male	11	55%	4	57%	15	45%	4	27%	0	0%	32	44%
Female	9	45%	3	43%	18	55%	11	73%	5	100%	40	56%
**Age**												
31–40	7	35%	0	0%	0	0%	4	27%	0	0%	10	14%
41–50	8	40%	0	0%	0	0%	9	60%	1	20%	12	17%
51–60	5	25%	0	0%	4	12%	2	13%	0	0%	11	15%
61–70	0	0%	0	0%	9	27%	2	13%	0	0%	9	13%
71–80	0	0%	0	0%	15	45%	2	13%	0	0%	15	21%
81+	0	0%	0	0%	5	15%	2	13%	0	0%	5	7%
Unknown	0	0%	7	100%	0	0%	0	0%	4	80%	10	14%

a *Includes four who also took part in the planning phase and 2 who took part in two interviews*.

b *Includes two who also took part in the planning phase*.

## Phase 1: Intervention Planning

### Methods

#### Design

The aim of intervention planning is to gather the information necessary to plan the intervention content and design. To achieve this, we conducted two qualitative interview studies to better understand the contexts and situations within which practitioners would access Empathico and the potential issues that may be perceived or encountered when seeking to adopt the behaviors suggested. This contextual information was considered alongside three literature reviews to identify and guide the design of relevant theory and evidence-based intervention components. Using this mixed-method approach increases the likelihood of (a) practitioners engaging with and successfully changing target behaviors and (b) the target behaviors having an important impact on health outcomes.

#### Literature Reviews to Identify Relevant Existing Evidence and Theory on Our Target Behaviors and Approaches to Changing Them

A recent systematic review and meta-analysis identified 28 studies that trained healthcare practitioners in clinical empathy and/or positive messages (24). We conducted a secondary analysis of the seven empathy interventions from that review, aiming to identify effective components of existing training to enhance clinical empathy for healthcare practitioners (40). We also conducted a secondary analysis of the 22 positive messages interventions from that review, aiming to identify effective ways of imparting positive messages that could be used by healthcare practitioners to communicate realistic optimism in clinical practice (41). Finally, we conducted a systematic meta-ethnographic synthesis of 26 qualitative studies which aimed to elucidate and compare patients' and clinicians' perspectives on communication within consultations for OA (42). This was important to ensure Empathico was relevant to interactions for OA in primary care.

#### Qualitative Interviews to Explore Primary Healthcare Practitioners' Perspectives on Training in Clinical Empathy and Realistic Optimism

Semi-structured telephone interviews with 16 General Practitioners [GPs], two nurse practitioners and two primary care physiotherapists explored their perspectives on communication skills training, clinical empathy, and realistic optimism, within the wider socio-cultural and economic context of clinical practice, in particular OA management in primary care. Interviews were conducted by SH, JV, and KS, and were transcribed verbatim and analyzed using thematic analysis (43).

#### Think Aloud Interview Study to Explore Practitioners' Perspectives on KEPE-Warm

Early in the intervention planning phase, we selected the KEPE-Warm intervention as a starting point for Empathico (see Patients' Perspectives) and transferred it from the original paper-based format to a web-based format. We conducted a think-aloud study to explore practitioners' immediate reactions to potential intervention content and identify barriers, misunderstandings and opportunities for improvement. Three GPs and 4 GP trainees were opportunistically recruited to take part in audio-recorded one-to-one face-to-face interviews. After obtaining informed consent, the interviewer (RT) helped the participant to practice speaking their thoughts out loud before asking them to navigate through the online intervention while verbalizing their thoughts. Interviews were transcribed and analyzed using a “Table of Changes” approach (37). This is a rapid method of analysis that codes positive and negative comments against each section of the intervention. We categorized interviewee comments and assessed them against several criteria (important to behavior change, in line with the Guiding Principles—see Integrating Findings to Develop Guiding Principles and Guiding Principles, repeated by multiple participants, easy/uncontroversial) to determine whether and what changes should be made to the intervention.

#### Using Findings to Plan the Intervention

The findings from the literature reviews and qualitative work were used to draft the intervention, and to develop guiding principles, a logic model, and a behavioral analysis.

##### Building the Draft Intervention

**Figure 2:**
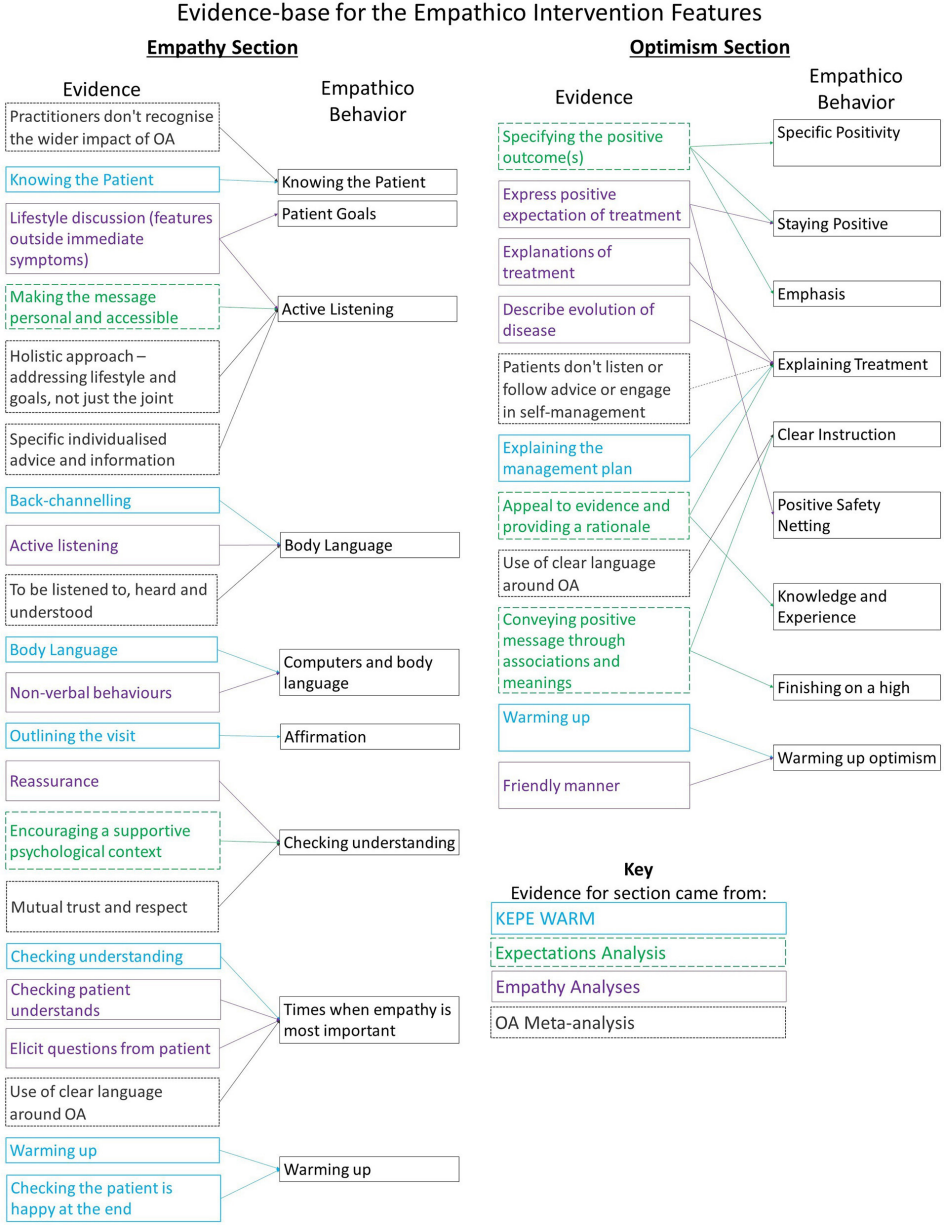
The evidence-base for the planned contents of Empathico training on clinical empathy and realistic optimism.

We used PowerPoint initially to draft content. We first designated each behavior a page, described the behavior and provided examples of the behavior. Where appropriate, Behavior Change Techniques were added to enhance the information (e.g., adding evidence from studies, and endorsements from other practitioners or from patients). An intervention flow diagram was created to show the information architecture of the intervention (see Figure 2 for the final version). The intervention draft was then implemented by KS in LifeGuide, an open source WYSIWYG (“what you see is what you get”) web application development tool designed for creating interventions for trialing (44).

##### Integrating Findings to Develop Guiding Principles

Guiding Principles are design objectives that the intervention must address to be optimally meaningful, relevant, acceptable, and practical for users specifying design features that will address those objectives. To devise our Guiding Principles, members of the multidisciplinary study team discussed study findings drawing on their experience of person-based digital interventions for health, professional experience in primary care consulting and PPI experience of OA. In this way, we identified key contextual or psychosocial issues likely to impact engagement with our intervention and specified how we would address these. We consulted and amended the Guiding Principles throughout planning and optimization as iterative feedback was received from end users.

##### Integrating Findings Into a Logic Model

The logic model is a visual representation that maps how the intervention is hypothesized to effect change in the intended outcomes. This helps researchers (1) to choose appropriate intervention components during planning and optimization and (2) to choose appropriate process measures during intervention evaluation. We developed the logic model based on the findings of our formal literature reviews and a broader reading of relevant literature and theory.

##### Using Findings in a Behavioral Analysis

The behavioral analysis (1) defines the target behaviors that the intervention seeks to change, including any necessary sub-behaviors and (2) identifies likely effective determinants of behavior change based on existing theory and evidence. Conducting a behavioral analysis supports transparent description of the intervention, encourages researchers to check the planned intervention is consistent with broader evidence and theory, and ensures there is sufficient and appropriate rationale for the inclusion of specific intervention components. We identified our target behaviors and necessary sub-behaviors with reference to our literature review work. We identified barriers and facilitators to performing these behaviors with reference to our literature reviews and qualitative interviews. We identified the likely effective determinants of these behaviors by characterizing them according to the COM-B model (5) as associated with the Capability of the practitioner to perform the Behavior, the practitioner's Opportunity to perform the Behavior, and the practitioner's Motivation to perform the Behavior. We used the Behavior Change Technique Taxonomy (a list of 93 behavior change strategies, e.g., goal setting, provision of information) (45) to specify behavioral techniques to incorporate into the intervention to support practitioners in changing their communication behaviors.

### Findings

#### Effective Components of Existing Training to Enhance Clinical Empathy for Healthcare Practitioners

Analysis of seven empathy trials examined three questions (1) which empathy behaviors were trained, (2) how they trained practitioners, and (3) which behavior change techniques (BCTs) were used. Eighteen empathy behaviors were identified–the most common were providing explanations of treatment, providing non-specific empathic responses (e.g., expressing understanding), displaying a friendly manner and using non-verbal behaviors.

We used the training methods and BCTs identified in the seven trials in our behavioral analysis. We found that the most used training approaches were face-to-face training (*n* = 5), role-playing (*n* = 3) and videos (self or model; *n* = 3). Of these, only videos were compatible with our chosen online format for our training. The BCT used most frequently to encourage practitioners to adopt empathy behaviors was “Instruction on how to perform behavior” (*n* = 5; for example, providing a video demonstration), followed by “Credible Source” (*n* = 4; for example, delivered by a medical professional) and “Behavioral Practice” (*n* = 3; for example, role-playing). We incorporated the first two but could not implement “Behavioral practice” within the online format of our intervention.

Of all the empathy interventions that we reviewed we chose to use the evidence-based “KEPE Warm” (46) as the initial basis for our Empathico intervention, because (a) the published pilot data from a randomized controlled trial involving 16 GPs and 190 patients suggested KEPE Warm effectively modified practitioner behavior and patient satisfaction, and (b) its brevity (15 min instruction and up to 1 h reflection) appeared to make it feasible for implementation in busy primary care settings, particularly compared to other interventions which took half a day or more or were developed for hospital or other non-primary care settings. Some members of the current research team had been involved in developing KEPE-Warm (PL, HE) and were able to share additional insights into its strengths and limitations.

KEPE-Warm was originally delivered in-person by a medical student who instructed GPs in 4 key behaviors: demonstrating **K**nowledge of the patient; **E**ncouraging the patient (e.g., through active listening); being **P**hysically **E**ngaging (e.g., though the use of appropriate touch and body language); **Warm**ing-up: being cool and professional initially, becoming warmer and more empathic during the consultation and avoiding non-verbal cut-offs at the end of the consultation. After the instruction GPs were asked to review videos of their own consultations collected previously and select three things they wanted to change about their behavior. KEPE-Warm incorporated most of the empathy behaviors and training techniques from the other effective interventions we reviewed but did not include instruction on learning the patient's goals and affirming their worries and concerns. We therefore added this content to our plan for Empathico. We further built on the framework of KEPE-Warm during intervention planning by adding additional evidence-based behaviors, transforming it into a digital format, and further optimizing it through the studies described in this paper.

#### Effective Ways of Imparting Positive Messages to Patients

Analysis of 22 expectancy interventions found five clusters of techniques for imparting positive messages: specifying the positive outcomes; making the message personal; drawing on associations and meanings; providing a supportive psychological context; and providing a rationale. Two of these clusters (“Making the message personal and accessible” and “Encouraging a supportive psychological context”) were a better fit conceptually with our planned Empathy section, and so techniques from these clusters were incorporated there instead.

We planned the contents of the Optimism section based on the three clusters not used in the Empathy section and focused on those techniques that could be achieved through practitioner communication behaviors within a consultation setting. This meant, for example, that we excluded techniques that required the immediate presence of a treatment (e.g., drawing attention to sensations, branding on packaging). This created 8 optimism elements, in addition to the “KEPE Warm” section on “Warming up” (increasing expressions of optimism toward the end of the consultation).

#### Patients' and Practitioners' Perspectives on Communication Within Consultations for OA

We synthesized 26 eligible qualitative studies to elucidate and compare patients' and practitioners' concerns and priorities regarding healthcare interactions for OA (47). The outcomes are summarized in Table 3. There were clear shortcomings in clinical communication about OA from patients' and clinicians' perspectives including a lack of perceived empathy, confirming the need for training on clinical empathy in relation to OA in particular. Patients and practitioners had discrepant understandings of OA and its management, supporting the need for better communication about the nature of the condition, its management, and likely treatment outcomes. Our meta-ethnography provided an in-depth understanding of patients' and practitioners' perspectives, in relation to each other, which enabled us to construct an OA section of the intervention that (1) Addressed discrepancies between patient and practitioner understanding (2) Provided a practical example of how the techniques described in the intervention could be applied and (3) Provided information and resources, both for practitioners and patients.

**Table 3:** Themes identified in the meta-ethnography.

	**Patients**	**Practitioners**
Priorities and Perspectives	To be listened to, heard and understood Mutual trust and respect Holistic approach—addressing lifestyle and goals, not just the painful joint Specific tailored advice and information Use of clear language when communicating about OA	Practitioners can normalize OA Uncertainty about what information OA patients need. Uncertainty about how to support self-management for OA
Concerns	OA not taken seriously by practitioners Practitioners don't recognize the wider impact of OA Practitioners are not experts in OA Unmet information needs about OA	Patients have variable and limited understanding of OA Patient expectations about OA are variable and unrealistic Patients need to be more informed about OA Patients don't listen or follow advice or engage in self-management Lack of time in the consultation

#### Primary Healthcare Practitioners' Perspectives

Our analysis of primary healthcare practitioners' perspectives on training in clinical empathy and realistic optimism identified multiple barriers and facilitators to engaging them in our training (see Table 4). Based on these findings, our intervention needed to: (1) address practitioners' concerns that incorporating clinical empathy and realistic optimism would increase consultation duration; (2) convey the importance of optimism being realistic in a clinical context; (3) address practitioners' concerns that expressing empathy would increase their risk of burn-out; (4) explain that clinical empathy can be communicated authentically without over-investment of emotional capital. These findings fed into the guiding principles and behavioral analysis and thus informed how we presented the intervention content.

**Table 4:** Barriers and facilitators to engaging with training, identified from practitioner interviews.

	**Realistic optimism**	**Clinical empathy**
Barriers to engaging with training	Practitioners talked about ”patient expectations” in terms of managing expectations rather than optimizing expectations. Need to be realistic when communicating about likely clinical outcomes with patients; should not encourage overly positive expectations that would be unachievable clinically. There is limited time in primary care consultations such that it might be difficult to “fit in” any additional, optimistic, communications. Optimism may sound unempathetic or hollow. Optimism might clash with fatalistic or otherwise negative patient expectations, which can be very firmly entrenched especially for long term conditions.	Practitioners believe empathy comes naturally or with experience rather than through instruction or training. Empathy can be difficult in some circumstances, e.g., with “difficult” patients. Fear that clinical empathy (as understood by practitioners, to include a felt-emotional component) increases risk of practitioner burn-out. Practitioners have already been trained in clinical empathy and may not feel they need more training.
Facilitators to engaging with training	Practitioners find empathy easier if they know the patient's expectations for the consultation The idea of being upbeat and positive in consultations is attractive. The idea of communicating realistic optimism is novel.	Practitioners believe that empathy is fundamental to consultations. Clinical empathy comes more readily when patient shows emotion, when patient is likable, and when the practitioner has personal experience with the condition.

#### Practitioners' Perspectives on KEPE-Warm

Analysis revealed that although the practitioners agreed that the advice in KEPE-Warm was valuable, there were several barriers to engaging meaningfully with the intervention content and subsequently adopting the recommended behaviors. For a full description of issues arising please see Supplementary Material 1. Barriers included poor information coherence (i.e., the information architecture was poorly organized so that it was unclear or unmemorable); familiarity (i.e., practitioners already knew the information so did not feel a need to re-engage with it); misunderstandings and disagreements (i.e., participants misunderstood or disagreed with some suggestions); and low feasibility (i.e., practitioners did not think they would be able to enact the behaviors in a typical consultation). We addressed these issues in two main ways. Firstly, we highlighed them in our Guiding Principles. For example, Guiding Principle 4 (Table 5) emphasizes the need to ensure behaviors learned in Empathico can be implemented without increasing practitioner workload including consultation duration. Secondly, we reworked problematic aspects of KEPE Warm when drafting the Empathico prototype. For example, participants did not understand the KEPE Warm acronym or find it easy to remember, and so we removed this from Empathico.

**Table 5:** Empathico guiding principles.

**Design objectives**	**Key (distinctive) intervention features**
1. To persuade practitioners to access and engage with the intervention (buy-in)	• Acknowledge previous expertise • Highlight benefits of engaging with the intervention • Provide evidence that a brief intervention can improve the consultation, even with very experienced GPs • Provide evidence that adopting behaviors from the intervention can make the consultation easier
2. To raise awareness that being realistically optimistic about treatments can improve (OA) patient outcomes.	• Provide placebo evidence • Provide evidence on patient experience/satisfaction—modeled into evidence-based “patient stories” • Provide clear explanation of what outcomes realistic optimism can support
3. To persuade practitioners of the benefits of using the things learnt from Empathico in all contexts (including challenging ones).	• Acknowledge frustrations and times when it may be difficult to employ the target behaviors • Provide clear evidence-based rationale (e.g., patients feel valued and heard; avoid misalignment of expectations) • Demonstrate respect for clinical judgement and acknowledge that some aspects of the toolkit may not be relevant in some contexts
4. To enable practitioners to communicate empathically and with realistic optimism without negatively impacting workload.	• Intervention must be simple, short and accessible • Core target behaviors must be memorable • Provide concrete examples of words, phrases or non-verbal behaviors that can be used • Suggest time-saving strategies e.g., reminder of existing resources that can be provided to the patient (booklets, weblinks) to support self-management
5. To motivate practitioners to acknowledge the wider impact of illness on the individual patient's daily life and well-being.	• Provide concrete verbal strategies for opening the consultation and eliciting patient expectations

#### Guiding Principles

The intervention Guiding Principles (Table 5) were developed primarily on the findings from the meta-ethnography and the primary qualitative research, as these studies provided the most direct evidence concerning intervention features that would facilitate engagement and should be included and those that might be a barrier to engagement and should therefore be avoided. Design objective (1) was introduced during the optimization phase of intervention development when the importance of buy-in became clearer.

#### Logic Model

The logic model was constructed in parallel with the other intervention planning work and is shown in **Figure 4**. On commencing our program of work, we had specified the problem we sought to address and our approach to accomplishing this—attempting to improve practitioners' communication of clinical empathy and realistic optimism (our intervention targets). Our literature reviews and behavioral analysis helped us to specify the other components of the logic model. The planned content of the intervention was summarized in the logic model (“intervention resources”) and was designed to effect change in practitioner behavior through the processes of increasing practitioner knowledge about clinical empathy and realistic optimism, increasing practitioners' beliefs that communicating clinical empathy and realistic optimism would benefit their patients (outcome expectancies), increasing practitioners' beliefs that they could better communicate clinical empathy and realistic optimism (self-efficacy), and increasing practitioners' skills and intentions to enact the new behaviors. These processes together are proposed to effect change in the patient's clinical outcomes and satisfaction with the consultation through several mediators. The first mediators are increased expressions of empathy and optimism by the practitioner, through which all the other mediators act. These influence patient perceptions of empathy and optimism and decrease patient anxiety. Perceived practitioner optimism increases the patient's perception that the treatment is credible and their response expectancy from the treatment.

#### Behavioral Analysis

We identified the following behaviors necessary to impact patient outcomes: the practitioner would need to complete the online training, video their consultations, reflect on their consultations, plan their behavior changes and enact empathy and/or realistic optimism behaviors in consultation. We extracted the barriers and facilitators from the studies reported above, with expert discussion and PPI input. We then identified the target constructs needed to address these barriers and the intervention functions. We then used the Behavior Change Taxonomy to identify appropriate techniques and describe the required intervention components. For example, our planning studies suggested that practitioners might forget to perform the new behaviors (a barrier), which suggested a need to support automatic motivation [from the COM-B model (5)], which can be addressed through environment restructuring [from the BCW (5)] using prompts or cues in the environment [from the BCT taxonomy (45)]; this analysis led us to develop Empathico post-it notes for practitioners to put on their desk as a cue to perform the new behaviors learnt through Empathico. See Supplementary Table 2 for a summary of the complete Behavioral Analysis.

#### Intervention Plan

**Figure 3:**
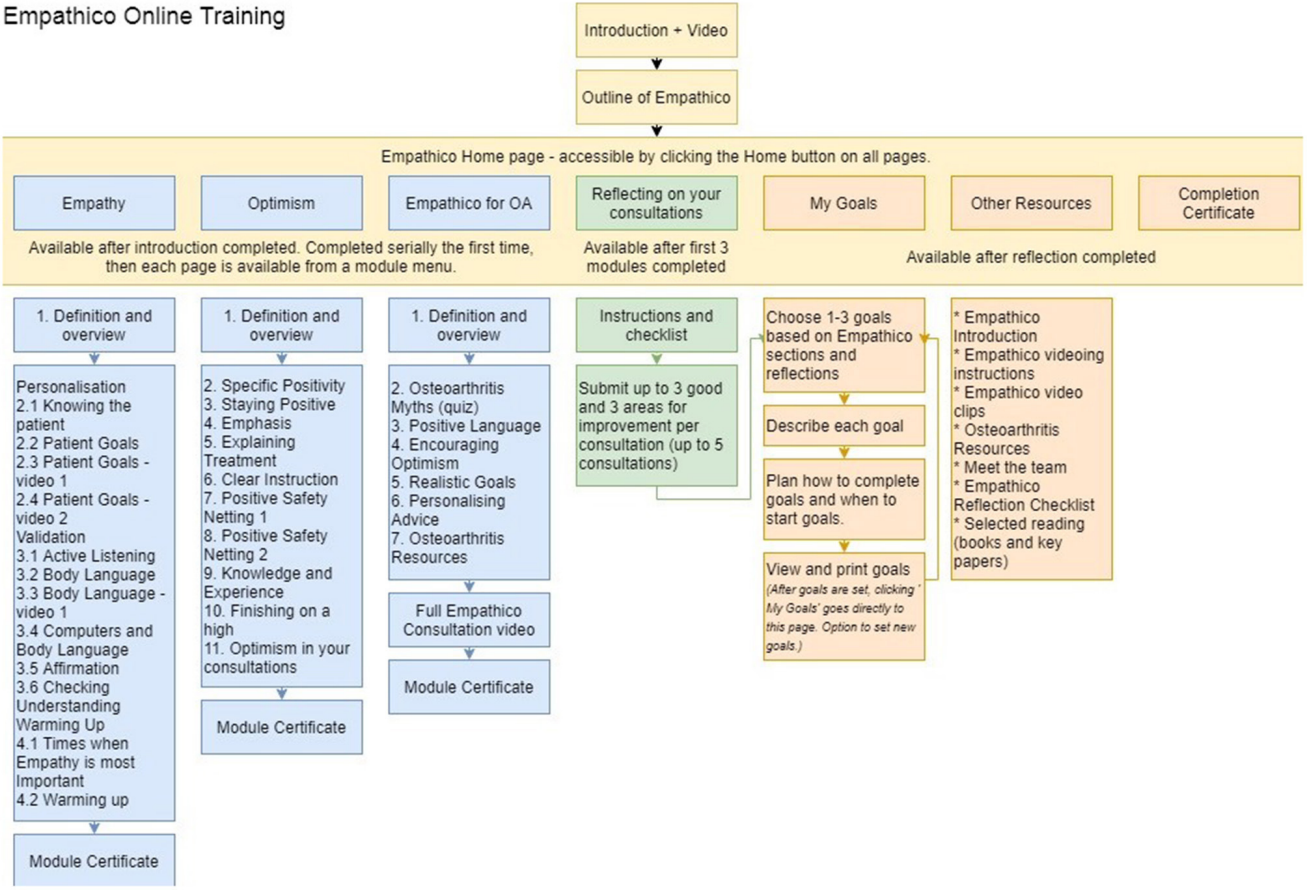
Overview of the Empathico structure and contents.

We called the intervention “EmpathicO—Improving care through Empathy and Optimism” (Empathico), as this title captured the core focus of our intervention using terminology that would be understood by practitioners without coming across as invalidating their existing knowledge and skills. Findings from our intervention planning work, including the guiding principles and behavioral analysis, were then integrated to formulate the overall structure and contents of Empathico. As depicted in Figure 3, the prototype intervention was divided into an introduction, three information sections, a reflection section, a goal-setting section and a resources section.

The introduction was designed to persuade users that the intervention is worth their time and effort by providing evidence for its efficacy and a brief persuasive introductory video from an authoritative and respected source (presented by co-author PL, a senior academic and GP). The introduction acknowledges the users' experience and provides an outline of the training and its evidence base.

The three informational sections focus on Clinical Empathy, Realistic Optimism and Applying Empathico in OA; these can be completed in any order, to give the user autonomy over their learning. Each of the information sections contain short paraphrased excerpts from patients and practitioners “Patients say…,” “GPs say…” or evidence boxes “Research shows…” with links to summaries of academic papers. These serve to persuade the user of the validity of the information. Each information section also has a module certificate at the end, and the user can review any of the material again after viewing it for the first time.

**Figure 4:**
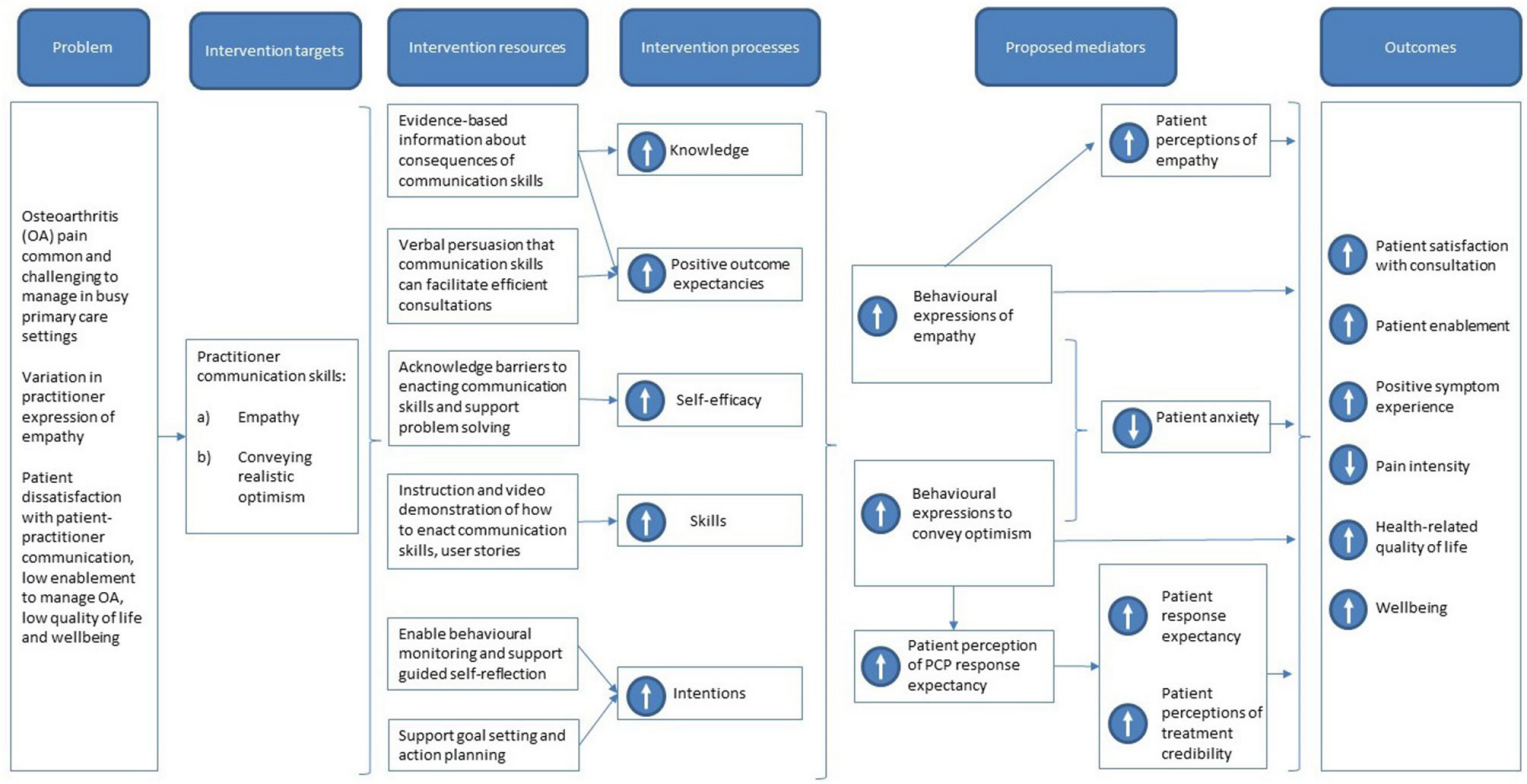
Logic model for Empathico.

The behaviors covered in the clinical empathy and realistic optimism sections are listed in Figure 4 which also depicts the source of the evidence to support their inclusion.

The empathy section acknowledges the users' prior knowledge and provides a definition of clinical empathy. It then presents details of verbal and non-verbal behaviors to communicate empathy to patients, as well as strategies for implementing them and examples of how they can be used in the context of primary care consultations. To further illustrate the contents, videos showing three of the target behaviors are provided.

The optimism section defines optimism and explains how studies of placebo effects demonstrate the power of positive messages to improve patients' health outcomes through known neuropsychological mechanisms. It then presents strategies for communicating realistic optimism to patients and gives examples of how this can be done within consultations. There are two short exercises for practitioners to identify ways they can make their consultations more optimistic.

The OA section contains a short quiz that addresses the misunderstandings between patient and practitioner beliefs. It presents strategies for addressing the specific challenges in communicating clinical empathy and realistic optimism in consultations about OA and provides examples of how these can be implemented. This section also includes an up-to-date treatment pathway for OA, links to OA resources for patients and practitioners and a film illustrating how Empathico behaviors can be integrated into a whole consultation about OA.

On completing the informational sections, the reflections section unlocks. This section directs users to review and reflect on video recordings of their own consultations that they made previously (instructions to record one's consultations were provided outside the main intervention). They are asked to do this with reference to a checklist of empathy and optimism behaviors covered in Empathico. The user is then prompted, for each consultation they have reviewed up to a maximum of five, to type into the intervention website between one and three things they did well and between one and three things they would like to improve on. On submitting these reflects the user is moved into the goal-setting section of the intervention.

In the goal-setting section, the user is directed to set up to three goals. Each goal should be to change one communication behavior, based upon their reflections and the Empathico material. For each goal, the user is instructed to plan when they will start the goal, i.e., when they will first attempt their planned behavior change, and to decide on a strategy to help remember the goal. “Empathico” branded sticky notes are supplied for this purpose.

On completing all sections, a completion certificate is made available for download. Further resources are made available for direct access from the main menu.

### Summary of Intervention Planning

Our three literature reviews and qualitative interview study effectively identified potentially effective intervention components, barriers and facilitators to practitioners engaging with the intervention, and features of OA consultations that required consideration. The draft guiding principles focused our intervention on the most important features and the behavioral analysis identified appropriate features to support communication behavior change in the context of primary care consultations. The logic model outlined how the intervention was hypothesized to impact patient outcomes. On completing intervention planning, we had a complete draft of our intervention.

## Phase 2: Intervention Optimization

### Methods

The aim of intervention optimization is to iteratively refine the intervention to ensure that it is optimally acceptable, motivating and feasible to use and adopt. To achieve this aim, we conducted three qualitative studies with primary healthcare practitioners and patients to provide rich data on how Empathico was perceived, reacted to and used in practice. Findings were used to iteratively modify the intervention and the underlying guiding principles. Using this iterative qualitative approach ensures barriers to engagement are addressed and increases the likelihood that the intervention will support behavior change.

#### Interview Study to Explore Patients' Perspectives

The aim of this study was to identify barriers and areas for improvement in the behaviors that Empathico teaches, from patients' perspectives. To convey to participants the behaviors that Empathico encourages practitioners to use, we scripted and filmed a model Empathico consultation and a neutral consultation, and wrote vignettes describing optimistic and neutral consultations. We showed the films to one set of participants (*n* = 15) and gave the vignettes to another set of participants (*n* = 18), all of whom had OA and were recruited from general practices. All patients then took part in a semi-structured one-to-one interview with a researcher (JV, EL) and the interviews were audio-recorded and transcribed verbatim. Thematic analysis was used to identify patterns in the data that summarized patients' perspectives on empathy and optimism in OA consultations [published elsewhere (48)]; findings were also analyzed specifically to help us create an intervention that was acceptable to patients, using the table of changes method.

#### Think-Aloud Interview Study With Practitioners

This study aimed to identify barriers to adopting behaviors encouraged by the intervention, technical errors, and areas for improvement in the intervention. Participants were recruited from General Practices in the South of England. Participants were primary care practitioners (GPs, nurses or physiotherapists) who consulted with OA patients. Participants could choose to take part at the participant's workplace, or at the University of Southampton. After giving informed consent, participants were presented with the intervention and asked to speak aloud their thoughts as they looked at it. The interviewers (JV, KS) prompted participants with questions (e.g., what are you thinking now?) if they stopped speaking and asked additional questions at the end to further explore their experience. The think aloud interview topic guide is in Supplementary Material 5.

#### Retrospective Interview Study With Practitioners

The aim of this study was to identify barriers, errors and areas for improvement in the intervention when used independently. Five participants were recruited from General Practices in the South of England. Participants had to be primary care practitioners (GPs, nurses or physiotherapists) and see OA patients to be eligible for the study. Participants were given a link to the study with a username and password. After giving informed consent, participants could look at Empathico whenever they liked over 2 weeks. Participants were not required to video record consultations prior to taking part. After 2 weeks, a telephone interview was arranged. The interviewer (JV) asked participants about their thoughts and experiences of Empathico.

#### Data Analysis Methods

Interviews in all three optimization studies were audio-recorded, transcribed and analyzed using the “Table of Changes” approach described above (section **Think Aloud Interview Study to Explore Practitioners' Perspectives on KEPE-Warm**) (37). In this phase, we made changes every 2–5 interviews, so that upon analyzing the next set of interviews, we could assess whether there was evidence for the change being effective. The table of changes method can also reveal key barriers, which allowed us to modify the intervention Guiding Principles. Interviews were conducted iteratively until no important issues were identified and the feedback was predominantly positive. A team of researchers contributed to the analysis, bringing perspectives from different disciplinary backgrounds including general practice (MR, EL, HE), primary care research (JV, SH), human computer interaction and digital interventions (KS, MS), health psychology (LM, RT, FB), and philosophy of science and epidemiology (JH).

### Findings

#### Patients' Perspectives

Patients were much more positive about the Empathico consultation than they were about the neutral consultation, regardless of whether they saw the filmed consultations or read the vignettes. Our table of changes analysis of patient interviews nevertheless highlighted some problems with the Empathico consultation, mainly in the form of omissions, and these are summarized in Table 6. Patients wanted the practitioner to have prior knowledge of themselves and their condition, they wanted their expectations to be acknowledged, and they wanted a clear and specific explanation of treatment and plan of action, and did not feel that the Empathico consultation fully met these needs. We therefore revised the Empathico intervention to ensure these points were incorporated.

**Table 6:** Problems with the Empathico consultation from patients' perspectives.

**Problem**	**Sample quote**	**Solution**
Practitioner did not explore the patient's expectations about treatment.	”I think the only person who knows your body is yourself - although I suppose, in my case, I could be completely wrong - but you think you do, and the assumption was that no surgical intervention was deemed necessary at this stage however correct that might be and it's those sort of possibilities that I would have like to know more about.“ (male, 61–70 yrs, knee OA)	Acknowledge patient's goal and expectations about treatment.
Lack of explanation for recommended treatment.	”She didn't go into […] the construction of the knee, and how if you can strengthen the muscles that are holding the knee in place. So she didn't fully explain. She just said these exercises will help the joints and muscles. I think she could have been far more explicit as to how important it is to strengthen the muscles holding the knee in place.“ (female, 71–80 yrs, hip and knee OA)	Where appropriate, explain underlying pathology and justification for treatment.
Balancing motivation with realistic outcomes.	”I suppose on reflection she perhaps could have pressed a bit more to try to motivate him a bit more, but then to try and motivate him you're probably going to give him a false expectation. If she makes too much of it, which motivates him, and it doesn't happen, that's worse. So it's six of one, and half a dozen of the other really.“ (male, 71–80 yrs, hip OA)	Ensure optimism is conveyed realistically and appropriately.
Practitioner didn't seem to know the patient's history	”The patient had to start at the beginning again and go through, which was not a good thing.“ (female, 71–80 yrs, hip and knee OA)	Recommendation to read patient notes prior to consultation.
No plan to review progress was made.	”[The doctor could have said] 'Let's do this 3 months, and let's come back and see me, and then we'll move forward;' rather than leaving it open-ended [.] That would give him much more confidence that he's been managed." (male, 51–60 yrs, hip OA)	Optimism about self-management, clear explanation (OA does not necessarily get worse), positive safety netting.

#### Practitioners' Perspectives on Intervention Components

Participants were mostly very positive about Empathico but multiple problems were identified and addressed particularly with earlier versions. Supplementary Table 3 presents examples of these problems, how they were raised by participants and how we addressed them. Proposed solutions took 1–2 iterations to optimize until the feedback on these sections was mostly positive and no further essential changes were identified through the table of changes analysis.

Problems were identified with the intervention in general (e.g., poor presentation on some cluttered pages, some omissions including strategies for dealing with difficult situations), and with the osteoarthritis section (e.g., instructions for the “Myths” quiz were unclear and an 8-min illustrative video was felt to be too long). Of particular interest were more conceptual problems identified with the empathy and optimism sections, illustrative examples of which are shown in Table 7 (for more, see Supplementary Table 3). Many of these problems highlighted the need to adapt our evidence-based recommendations about communication behaviors to make them more appropriate for implementation by primary healthcare practitioners.

**Table 7:** Illustrative examples of problems and solutions identified through “table of changes” analysis of think-aloud interviews on intervention components.

**Intervention section**	**Problem**	**Sample quote**	**Solution**
Empathy	Practitioners didn't like being told to act with “authority and professionalism.”	“'I'm not sure whether, how people would feel about kind of changing to act with more authority and professionalism at the beginning of a consultation. I think most GPs would kind of, expect to be acting professionally all the way through the consultation, not just at the beginning, all the way through. And acting with authority… I'm not really sure what that means.” (male GP, 31–40 yrs)	Remove this phrasing, change to emphasize increasing empathy throughout the consultation.
Empathy	Belief that “knowing the patient” takes time that is not always available.	“trying to make the time to add that in is actually really challenging and it's how we would all love to be working as GP's because it makes, it does help the consultation it everything more rewarding it does feel a much more natural way to communicate but I think time is the big barrier to that.” (female GP, 41–50 yrs)	Reassure them that it doesn't have to add time, and provide examples.
Empathy	Patient goals are not always appropriate.	“Patient's goals can be wide and nebulous and difficult to come back to.” (female GP, 41–50 yrs)	Provide a strategy to help practitioners help patients formulate realistic goals.
Empathy	Practitioners uncertain about avoiding use of non-verbal cut-offs to close a consultation.	“that might sometimes include standing up and, you know, walking the patient, in a nice way, toward the door. Sometimes. So yeah, I think it might be a bit over… over-simplifying the situation” (male GP, 31–40 yrs)	Remove directions to avoid “non-verbal cut-offs” and provide strategy for finishing the consultation empathically.
Optimism	Disagreement with advice to be “concrete” about treatment outcomes.	“'Research says – Being concrete and specific about treatment options……' I am not usually very concrete about this. You can't say it's going to get better if you leave it alone – it might not! You can say it probably will get better and lets see how it goes but you can always come back – that sort of thing.” (female GP, 41–50 yrs)	Reword advice to talk about being specific when possible about expected outcomes.
Optimism	Practitioners uncertain about using the term “strong” or “potent” to describe a drug.	“Under the qualities of treatment I probably would refrain from using this as a strong drug just because in my experience, if you tell patients that something's very strong, then they worry about side effects, and they worry about it's too strong for them! Especially with the elderly patients, they want just something gentle that works” (male GP, 31–40 yrs)	Advise practitioners to use the terms when they are appropriate.
Optimism	Practitioners cautious about suggested phrases for “positive safety netting.”	“sometimes you have to say if it gets worse (eg acute chest infection). Need to be careful that patients take getting worse seriously.” (female GP, 31–40 yrs)	Make sure examples are appropriate for serious conditions, and that they are examples that don't fit all situations.
Optimism	Practitioners felt optimism is not always possible in challenging situations.	“the patient who is very negating of everything that you're suggesting, it might be something like, ‘I know this is difficult but I'm hoping you're gonna-, I think we can come up with a plan, I hope that you're feeling positive about it too’. Because then they can say ‘well not really,’ and then you're back to square one.” (female GP, 51–60 yrs)	Acknowledge that it is not possible in all situations.

Some recommendations conflicted with practitioners' beliefs or practice, such as the suggestion within the empathy section to act with “authority and professionalism” at the beginning of consultations; in this case, we removed the suggestion to act with “authority and professionalism” and instead emphasized the need to increase one's communication of empathy as the consultation progresses. The optimism section included material about “positive safety-netting” a phrase we used to refer to framing conversations about safety-netting positively (e.g., “If you feel that it isn't right for you…” promotes autonomy to decide if they like treatment) instead of negatively (e.g., ”If that doesn't work…“ suggests treatment might not be effective). This was a novel suggestion for practitioners and there was some concern about how this might risk patients not taking seriously any symptom exacerbations; we therefore added some additional guidance on positive safety-netting.

Some recommendations were felt to be overly simplistic to be of use in primary healthcare consultations. For example, eliciting and later referring back to patients' goals (within the empathy section) was felt to be challenging when patients have vague and/or unachievable goals; we addressed this by adding content on how to guide patients to formulate realistic goals. In the optimism section we had suggested using terms such as “strong” or “potent” to describe a prescribed drug (based on our review of positive message interventions). Practitioners were concerned that this might not always be an accurate description of prescribed medication and might be off-putting for some patients; we amended our guidance to remove the term “potent” and presented “strong” as an example of one way to communicate realistic optimism that could be used where appropriate.

#### Practitioners' Perspectives on the Whole Intervention

Feedback from the retrospective interviews was mostly positive, which was to be expected given the changes we had already made to address issues uncovered by the think aloud interviews. Very few problems with the empathy or optimism sections were identified at this stage. Most problems related to easily fixed technical problems or omissions, some of which came to light because—in contrast to the think aloud studies–practitioners in this study had been asked to work through the entire intervention in their own time. For example, participants wanted to see their progress through the intervention and so progress “breadcrumbs” were added to pages. The retrospective interviews also identified problems with the osteoarthritis and reflections and goal-setting sections. For example, the reflections that practitioners typed into the website could be lost if not saved, and so a “save” button was added to this page. Some problems were not acted on because they were impossible to address or were considered highly unlikely to act as a barrier to engagement with the intervention. Table 8 presents illustrative examples of problems and solutions; Supplementary Table 4 presents more examples.

**Table 8:** Illustrative examples of problems and solutions identified through “table of changes” analysis of interviews with practitioners who had tried Empathico.

**Intervention section**	**Problem**	**Sample quote**	**Solution**
General	Practitioners struggled to print/save the certificate.	“Big problems printing out the certificate. Had to copy and paste to a separate word document. Would normally just download and attach electronically to appraisal.” (female GP, 41–50 yrs)	Provide instructions on how to save/print the certificate.
General	Practitioners wanted more detail on how to handle challenging situations.	“Yes. So sometimes the more your patients might be a bit challenged, you find it challenging with communication. So if you feel that there's a barrier to that, whether that's English isn't a first language, or culturally, or just you don't feel that they've necessarily got a level of comprehension, I find that difficult.” (female physiotherapist, 13 years' experience)	Added challenging situations page.
Osteoarthritis	Not enough diversity in videos.	“Could have had another example. Just used the same bloke all the way through. Might add variety of someone with OA in a different joint (shoulder/hand etc). Have a couple of different scenarios might enable people to reflect further.” (female GP, 41–50 yrs)	No other videos available—no change. Review in future if resource becomes available to create additional clips.
Reflections and Goal setting	Practitioners think the reflection and goal setting take too much time.	“I think that's helpful, but realistically we're time-poor, so we might not necessarily do that.” (female nurse practitioner, 19 years' experience)	Nothing—this is already brief. Will investigate further in the feasibility trial.

### Summary of Intervention Optimization

In this phase we began with a plan of intervention content and components for behavior change developed using the PBA. We prototyped the intervention and iteratively improved it using think-aloud interviews with end users. We developed model Empathico consultation videos and written vignettes and obtained feedback from patients. We tested the intervention by giving it to participants and letting them use it alone, making final improvements based on feedback. This iterative approach allowed us to make significant improvements to the Empathico intervention to maximize its potential efficacy and acceptability to practitioners.

## Discussion

This paper described the planning and development of the Empathico Intervention using a person-, evidence- and theory-based approach. By involving target users at all stages of development, and using a systematic approach to refining it, we have maximized the potential of the intervention to be effective. This focus on user engagement is particularly valuable when trying to implement evidence from the placebo literature into clinical practice, an endeavor that is often met with valid ethical concerns as well as objections founded on misunderstandings and myths about placebo effects (49, 50).

Other digital interventions for patients with OA typically aim to support rehabilitation and improve patient self-management [e.g., see interventions reviewed in (51)]. Empathico is different in that it targets those practitioners who treat patients with OA in primary care settings, and aims to enhance their communication skills for use in practitioner-patient conversations about many different forms of treatment (including, for example, pain medications, exercise, and even patient-facing digital interventions to support self-management).

There are some limitations to our work. Due to the time necessary to analyze rich qualitative data, the number of participants involved in the development was not high enough to ensure minority representation. Only 3 of our 72 participants were from non-White ethnic backgrounds (Table 2), meaning we may have missed opportunities to learn about the specific challenges and opportunities for communicating with people of different ethnicities. The practitioners involved in our study were self-selecting in that they signed up to take part in a study on empathy training, and in interview all agreed on its value. The beliefs and opinions of practitioners who do not value empathy (who arguably would benefit from the training most) were not represented. We also interviewed mostly senior GPs—junior GPs, nurses and physiotherapists were under-represented, and their training needs might be different.

Empathico would benefit from two final development activities: integrating advice for communicating clinical empathy and realistic optimism with patients from diverse, Black, Asian, and other non-White ethnic minority backgrounds; and integrating advice for communicating clinical empathy and realistic optimism when consulting with patients over the telephone or on video calls. The next step is to test Empathico in a feasibility trial to determine how best to assess its efficacy (including which outcomes to measure using which instruments), and then to move on to a fully powered RCT to assess whether using Empathico to train practitioners in Clinical Empathy and Realistic Optimism can have an impact on patient satisfaction, health and well-being.

## Data Availability Statement

The datasets generated for this article are not readily available due to ethical considerations; making the data available would breach confidentiality of participants in our qualitative studies. Requests to access the datasets should be directed to Felicity L. Bishop, F.L.Bishop@southampton.ac.uk.

## Ethics Statement

The studies involving human participants were reviewed and approved by National Research Ethics Service West Midlands-South Birmingham Research Ethics Committee. The patients/participants provided their written informed consent to participate in this study.

## Author Contributions

LM, JB, JH, CM, PL, GL, HE, and FB: conceptualization. KS, JV, LM, SH, JB, JH, PL, MR, EL, GL, HD-M, HE, and FB: methodology. KS, SH, and MS: software. KS, JV, LM, SH, MS, RT, JH, MR, EL, PM, HE, and FB: formal analysis. KS, JV, SH, MS, RT, EL, HE, and FB: investigation. KS and FB: writing – original draft. JV, LM, SH, MS, RT, JB, JH, CM, PL, MR, EL, PM, GL, HD-M, and HE: writing – review and editing. KS: visualization. LM, JH, CM, PL, GL, HE, and FB: supervision. HE and FB: project administration. LM, JH, CM, PL, GL, HE, and FB: funding acquisition. All authors contributed to the article and approved the submitted version.

## Conflict of Interest

CDM has supported BMS to recruit to a non-pharmacological atrial fibrillation trial. The authors declare that the research was conducted in the absence of any commercial or financial relationships that could be construed as a potential conflict of interest.

## Publisher's Note

All claims expressed in this article are solely those of the authors and do not necessarily represent those of their affiliated organizations, or those of the publisher, the editors and the reviewers. Any product that may be evaluated in this article, or claim that may be made by its manufacturer, is not guaranteed or endorsed by the publisher.
