# Supplementary material for: Harnessing Placebo Effects in Primary Care: Using the Person-Based Approach to Develop an Online Intervention to Enhance Practitioners' Communication of Clinical Empathy and Realistic Optimism During Consultations
Source: Front Pain Res (Lausanne). 2021 Aug 24;2:721222. doi: 10.3389/fpain.2021.721222 (PMC8915751; doi:10.3389/fpain.2021.721222)
Supplement: Supplementary file 1 [file Data_Sheet_1.PDF]

Supplementary Material

Contents

1.1 Supplementary Material Table 1: Barriers identified from the think-aloud interviews of KEPE Warm 1

1.2 Supplementary Material Table 2: Behavioral Analysis of Empathico Behaviors 3

1.3 Supplementary Material Table 3: 'Table of Changes' results of the think-aloud interviews on intervention components........ 8

1.4 Supplementary Material Table 4: 'Table of Changes' results of the retrospective interviews with practitioners who had tried Empathico........ 14

1.5 Supplementary Material 5. Topic Guide for 'Think-aloud interviews' 17

1.1 Supplementary Material Table 1: Barriers identified from the think-aloud interviews of KEPE Warm

Category	***Description***
Alienating	Specific phrasing of the advice could alienate users and imply they were not already acting professionally
Coherence	Advice did not appear to be always consistent or would benefit from being presented in a different sequence.
Evidence	Additional evidence on which the intervention content was requested.
Flexibility	A need to be flexible (rather than prescriptive) about how the advice is implemented. Participants also commented that particular techniques may be appropriate/inappropriate or work more/less well in specific contexts.
I do this already	Some information was described as too basic, patronizing or something they do know. Known information also acknowledged to be reassuring, not all participants agreed as to which aspects of the intervention were known vs. new.
Intervention focus unclear	Unclear what the focus of the intervention was, or what the KEPE Warm acronym stood for.
Memorability	Some pages were described as conveying a lot of information, not always in keeping with the KEPE Warm acronym. One participant

	appreciated opportunity to revisit pages. Some suggested the information would be more memorable if it followed a sequential order (mimicking the context and chronological process of the consultation).<br><br>BUT: generally, participants liked the idea of having an acronym to hang the information on.
"Should" language	Avoiding 'should' language which implies prescription (see flexibility), and also that certain suggestions are optional when in reality they have to be done (e.g. explaining management plan)
Skills cannot be taught	Some communication skills cannot be taught – GPs have a natural ingrained style of consulting that is unlikely to change after using the intervention.
Topic specific context	Interventions were too general and may benefit from focusing on OA specifically or other clinical condition.
Unfamiliar phrases	Some terminology used in the intervention was unfamiliar or unclear. Key examples were: "social talk/social conversation/social history", "self-disclosure", "back channeling", "dispelling nervousness".
Unrealistic	Advice was good, in an ideal world, but unrealistic within the context of a time-constrained primary care consultation.
'Warming up' counterintuitive or contradictory	Being cool and professional at the start contradicts other advice present in KEPE-Warm e.g. to engage in social talk. A cool and professional start could be contrary to a PCPs natural style and some questioned how useful that would be for patients, particularly those already known to the GP.

1.2 Supplementary Material Table 2: Behavioral Analysis of Empathico Behaviors

**Target Behavior**	**Barriers and facilitators to target behavior, from practitioners' perspectives ***	**Target constructs from COM-B model**	**Intervention functions from BCW**	**Behavior Change Techniques from BCT taxonomy v1**	**Empathico components**
**Complete online training**	Barriers: lack of time; feel they are already trained; perceive empathy comes naturally (or not) rather than being amenable to training.<br><br>Facilitators: topic of optimism is novel; training contributes to CPD [continuing professional development].	Motivation (reflective)<br><br>Capability (physical)	Persuasion<br><br>Incentivization<br><br>Enablement	9.1 Credible source<br><br>5.1 Information about health consequences<br><br>5.3 Information about social and environmental consequences<br><br>5.6 Information about emotional consequences<br><br>6.3 Information about others' approval<br><br>10.1. Material incentive (behavior)<br><br>10.2. Material reward (behavior)	Sections are short<br><br>Timing for each section is clear<br><br>Contributes to professional development (CPD)<br><br>Information is novel<br><br>Logging in and out to stop and start is easy<br><br>Webpages are easy to navigate<br><br>Certificates can be downloaded upon completion

**Video-record consultations**	Barriers: lack of time; concerned patients may be reluctant to be videoed; administrative burden e.g. around consent and data security; difficult to set up.<br><br>Facilitators: potentially helpful for learning new skills	Motivation (reflective)<br><br>Capability (physical)<br><br>Capability (psychological)	Persuasion<br><br>Education<br><br>Training<br><br>Enablement	4.1 Instruction on how to perform a behavior<br><br>6.1. Demonstration of the behavior<br><br>9.1 Credible source<br><br>5.1 Information about health consequences<br><br>5.3 Information about social and environmental consequences<br><br>5.6 Information about emotional consequences<br><br>6.3 Information about others' approval	Clear instructions on how to record consultations, including consent and data protection issues<br><br>Physical and technical support from researchers<br><br>Evidence for effectiveness of recoding and reflecting on consultations<br><br>Provide recording equipment<br><br>Provide supporting documents (patient information sheets and consent forms)
**Reflect on videos of consultations**	Barriers: Technology; lack of time; reluctant to watch oneself<br><br>Facilitators: potentially helpful for learning new skills	Motivation (reflective)<br><br>Capability (physical)<br><br>Capability (psychological)	Persuasion<br><br>Training<br><br>Enablement	4.1 Instruction on how to perform a behavior<br><br>6.1. Demonstration of the behavior	Clear instructions<br><br>Evidence for effectiveness of reflecting on videos<br><br>Reassure users that reflecting on videos

				9.1 Credible source<br><br>5.1 Information about health consequences<br><br>5.3 Information about social and environmental consequences<br><br>5.6 Information about emotional consequences<br><br>6.3 Information about others' approval	need not be overly time consuming<br><br>Checklist of specific things to reflect on
**Plan to change consultation behaviors**	Barriers: forgetting to perform the new behaviors; lack of time to perform the new behaviors; too tired to perform the new behaviors.<br><br>Facilitators: performing the new behaviors would improve consultations	Motivation (reflective)<br><br>Motivation (autonomic)<br><br>Opportunity (environment)	Persuasion<br><br>Environment restructuring	1.1. Goal setting (behavior)<br><br>1.4. Action planning<br><br>1.5. Review behavior goal(s)<br><br>2.3. Self-monitoring of behavior<br><br>7.1. Prompts/cues	Website sends email reminders to users<br><br>Provide example plans<br><br>Section on goal setting and planning<br><br>Empathico post-it notes to put on desk as a cue/prompt to perform new behaviors

				8.3. Habit formation	Users can print out their personal goals
**Engage in empathy behaviors**	Barriers: time pressures in primary care consultations and surgeries; patient characteristics may make empathy feel difficult; need to use computers during consultations to read/make notes; speaking a different language; patient illness; personal prejudices (e.g. having a different socio-economic background); patient emotion (e.g. anger); expressing empathy can feel fake; don't want to use up a limited pool of empathy; don't want to be too empathic or risk burnout; empathy is a natural disposition not a skill that can be taught; empathy is not necessary for all conditions.<br><br>Facilitators: support from others (e.g. colleagues); communicating empathy provides benefits for patients and practitioners; some empathy behaviors are novel; it is easier to be empathic if the practitioner has personal experience of the patient's condition; patient characteristics may make empathy feel easy; recognizing patients' emotions helps one to communicate empathy.	Motivation (reflective)<br><br>Capability (psychological)	Persuasion<br><br>Education<br><br>Modelling<br><br>Training	4.1 Instruction on how to perform a behavior<br><br>5.1 Information about health consequences<br><br>5.3 Information about social and environmental consequences<br><br>5.6 Information about emotional consequences<br><br>6.1. Demonstration of the behavior<br><br>6.3 Information about others' approval<br><br>9.1 Credible source	Instruction on how to perform the empathy behaviors<br><br>Persuasion to overcome barriers<br><br>Demonstration of empathy behaviors (textual examples, videos)<br><br>Evidence (e.g. quoting studies that show its effectiveness)<br><br>Reassurance that performing empathy behaviors need not increase consultation duration
**Engage in optimism behaviors**	Barriers: time pressures in primary care consultations and surgeries; speaking a different language; patient characteristics may make optimism	Motivation – reflective	Persuasion, education, modelling, training	4.1 Instruction on how to perform a behavior	Instruction on how to perform optimism behaviors

	difficult; patient illness may make optimism difficult or inappropriate.<br><br>Facilitators: practitioner attitude; knowing the patient makes it easier to communicate realistic optimism; awareness of placebo effects makes it easier to communicate realistic optimism; novelty of optimism behaviors.	Capability - psychological		5.1 Information about health consequences<br><br>5.3 Information about social and environmental consequences<br><br>5.6 Information about emotional consequences<br><br>6.1. Demonstration of the behavior<br><br>6.3 Information about others' approval<br><br>9.1 Credible source<br><br>13.2 Framing/ reframing	Persuasion to overcome barriers<br><br>Demonstration of optimism behaviors (textual examples, videos)<br><br>Evidence (e.g. quoting studies that show its effectiveness)<br><br>Reassurance that performing optimism behaviors need not increase consultation duration

Note. * Evidence of barriers and facilitators came from the practitioner interview study (for all target behaviors), the meta-ethnography (all target behaviors), and the KEPE Warm study think aloud interviews (for engage in empathy behaviors, reflect on videos, and complete online training).

1.3 Supplementary Material Table 3: ‘Table of Changes’ results of the think-aloud interviews on intervention components.

Section	Problem	Sample Quotation	Solution
General	The intervention was presenting content that the practitioners already knew.	0201: “it’s a bit frustrating, rehashing stuff that is, for me, like reminding me what the alphabet is.”	To present the content as an opportunity to refresh and reflect on current practice.
General	Practitioners don’t want to take the time and paperwork to video their consultations.	0203: “Trying to get a doctor, a busy GP to video consultation, it would be quite a task.”	To provided evidence of its efficacy from an authority source.
General	Wanted to hear the patient perspective.	0203: “actually there might be some value in just putting in a clip of saying, you know, “I really like this doctor because they did this, this and this and this is what it meant for me.” I think that would be quite powerful.”	To provide patient quotes supporting the message
General	Wanted a strategy to deal with difficult patients.	0213: “at the beginning it says, 'Empathy can be most useful when you've got a difficult patient', and that's the bit that I think I find difficult, and I think probably most people find difficult, and the module doesn't really address that, how can you show empathy and feel empathy with a difficult patient? I think that's what would be really useful.”	Provide a strategy for difficult situations
Introduction	Formatting of the text was not optimal.	0204: “Ok interesting choice of what you have put in bold… ok. I think if I was doing it, I’d have highlighted the ‘patient satisfaction’ and ‘health outcomes.’”	Format the text so it is emphasized the most important points.

Introduction	Evidence box was not being looked at	0202: "About the evidence box: it adds another layer. If you try to get information across quickly, it's something else to read. And on the first slide, I just didn't look at it at all. I've only looked at now that you've brought my attention to it."	Make the evidence box stand out more by changing it to a contrasting colour
Empathy	Practitioners didn't like being told to act with 'authority and professionalism'	0201: "'I'm not sure whether, how people would feel about kind of changing to act with more authority and professionalism at the beginning of a consultation. I think most GPs would kind of, expect to be acting professionally all the way through the consultation, not just at the beginning, all the way through. And acting with authority… I'm not really sure what that means."	Remove this phrasing, change to emphasize Increasing empathy throughout the consultation.
Empathy	Practitioners found the explanation of validation unclear	0202: "I find it difficult to understand. I understand the word validation. It's about expressing empathy. So you're actually expressing empathy and understanding to a patient's position? I don't feel like the English is very clear there."	Provided explanation: The aim is not to validate that their condition is serious, but to acknowledge the concern that has brought them to consult you, regardless of the condition.
Empathy	Practitioners were confused by 'warming up'	0211: "I would have thought warming up is the first thing, like in sport, warming up, the warming up at the end, I would probably put it to the first, because you're thinking sprinters, what do they do first to not tear their muscles and tendons, they warm up first. That feels to me the wrong way around, that's why I'm struggling a little bit with warming up, because that's the first thing, you say hello to the patient and warm up	Change 'warming up' to 'increasing expressions of empathy', provide evidence

		that way, that's what I would understand as warming up.”	
Empathy	Practitioners objected to some of the examples.	0202: “I personally wouldn't use this sentence.’ Have you been busy since I last saw?’ You know, I don’t think busy-ness is what we want to be talking about. Because we want to be talking about health. So I wouldn't use that.”	Remove some examples, add others in.
Empathy	Belief that ‘knowing the patient’ takes time that is not always available	0204: “trying to make the time to add that in is actually really challenging and it’s how we would all love to be working as GP’s because it makes, it does help the consultation it everything more rewarding it does feel a much more natural way to communicate but I think time is the big barrier to that.”	Reassure them that it doesn’t have to add time, and provide examples
Empathy	Patient goals are not always appropriate	0202: “Patient’s goals can be wide and nebulous and difficult to come back to.”	Provide a strategy to help practitioners help patients formulate realistic goals
Empathy	Exercises are too simple, practitioners felt patronised	0201: “It seemed a bit too simplistic and basic, because it's quite like a comprehension test”	Remove the exercises
Empathy	‘Appropriate touch’ was not thought to be good advice.	0201: “inappropriate physical contact such as the pat on the shoulder, I’m not sure how appropriate that is, it really depends on your relationship with the patient, and the dynamics of consultation. As a 39-year-old man, patting a 17-year-old girl on the shoulder if she's come in complaining of knee pain, I’m not sure that would be appropriate. You might need to have a look at that.”	Remove advice on ‘appropriate touch’

Empathy	Practitioners uncertain about use of non-verbal cut-offs.	0201: “that might sometimes include standing up and, you know, walking the patient, in a nice way, towards the door. Sometimes. So yeah, I think it might be a bit over… over-simplifying the situation”	Remove directions to avoid ‘non-verbal cut-offs’ and provide strategy for finishing the consultation well.
Optimism	Definition of placebo not right	0205 “’Experts say – a placebo is any treatment that has no active effect on health’ – oh that is interesting. Well it does have an effect. I would disagree with that bit there – there has been loads of studies that show that people do get better on placebos.”	Reword placebo definition
Optimism	Repeated use of the same evidence	0212: “I'm just reading the little cross box of observational study about listening, supportive listening approach without injecting artificial optimism too early. This is the same study that we were looking at before which talked about the coolness at the start and warmth at the end, I think”	Use more varied evidence
Optimism	Disagreement with advice to be ‘concrete’ about treatment outcomes	0205: “’Research says – Being concrete and specific about treatment options……’ I am not usually very concrete about this. You can’t say it’s going to get better if you leave it alone – it might not! You can say it probably will get better and lets see how it goes but you can always come back – that sort of thing.”	Reword advice to talk about being specific when possible about expected outcomes
Optimism	Practitioners uncertain about using the term ‘strong’ or ‘potent’ to describe a drug.	0212: “Under the qualities of treatment I probably would refrain from using this as a strong drug just because in my experience, if you tell patients that something's very strong, then they worry about side effects, and they worry about it's too strong for them! Especially with the elderly	Advise practitioners to use the terms when they are appropriate.

		patients, they want just something gentle that works”	
Optimism	Keep the focus on OA when examples are used.	0208: “I wouldn’t bring asthma into the equation at this stage. I would try and find another OA example. Feels totally off-topic.”	Consistently use OA examples
Optimism	Practitioners cautious about suggested phrases for ‘positive safety netting’	0209: “sometimes you have to say if it gets worse (eg acute chest infection). Need to be careful that patients take getting worse seriously.”	Make sure examples are appropriate for serious conditions, and that they are examples that don’t fit all situations
Optimism	Practitioners thought that the ‘Knowledge and Experience’ section conflicted with a collaborative patient-centered approach	0208: “Quite paternalistic Should try and make it sound like a more collaborative approach. That the patient has had some sort of choice.”	Reword the text to be explicitly patient-centered.
	Practitioners felt optimism is not always possible in challenging situations.	0207: “the patient who is very negating of everything that you’re suggesting, it might be something like, ‘I know this is difficult but I’m hoping you’re gonna-, I think we can come up with a plan, I hope that you’re feeling positive about it too’. Because then they can say ‘well not really,’ and then you’re back to square one.”	Acknowledge that it is not possible in all situations
Osteoarthritis	Practitioners did not understand the task	0213: “so hang on, am I supposed to say whether it's true or false that this is a myth, or whether the statement is true or false?”	Provide clearer instructions.

Osteoarthritis	Practitioners did not appreciate 'trick' questions	0208: "'OA gets worse with increasing age.'… What you say there doesn't really fit. Yes of course things flare up and settle down, but in general, you don't have OA in your 20s, a bit when you are 40, a lot when you are 60 and a lot more when you are 80. You would struggle to find evidence that this is not true. It doesn't mean the patient has to feel hopeless about the future."	Change the phrasing of the question to fit the evidence more accurately.
Osteoarthritis	Practitioners did not like 'Wear and Repair' reframing of OA	0207: "when I say wear and repair, I sort of think that… if you've got somebody with a hip arthritis which shows it's bone on bone, that isn't going to repair itself, so I worry that that is slightly falsely optimistic"	Remove 'Wear and Repair' and provide justification and evidence.
Osteoarthritis	Uncertainty about clinical recommendations	0207: "I'm surprised COX 2 inhibitors are still on there"	Provide clear reference to national recommendations with date of access.
Osteoarthritis	OA video too long	0213: "The fact that it's eight minutes. Well, you know, if I'd set aside some time to do this, then that would be fine, if I'm trying to fit it in at the end of a busy day I might not, I might skip it."	Provide options to skip it and view it later.

1.4 Supplementary Material Table 4: 'Table of Changes' results of the retrospective interviews with practitioners who had tried Empathico.

Section	*Problem*	*Sample Quote*	Solution
General	Practitioners struggled to print/save the certificate	0403: "Big problems printing out the certificate. Had to copy and paste to a separate word document. Would normally just download and attach electronically to appraisal."	Provide instructions on how to save/print the certificate
General	Practitioners would value a summary sheet.	0401: "I suppose, maybe at the end of each section, a sort of bullet point list to print off, of things to remember."	Added a contents page to each module with summary of content
General	Practitioners wanted to see progress through the section	0404: "I didn't know how far through each section I was, so if there was a way to put on there, like I don't know, you're on slide eight of 12, or you're 60 per cent of the way through this section, I would've found that quite useful, probably."	'Breadcrumbs' (dots that indicate page progress) added to the intervention
General	Practitioners wanted more detail on how to handle challenging situations	0406: "Yes. So sometimes the more your patients might be a bit challenged, you find it challenging with communication. So if you feel that there's a barrier to that, whether that's English isn't a first language, or culturally, or just you don't feel that they've necessarily got a level of comprehension, I find that difficult."	Added challenging situations page
Introduction	Video audio was too quiet	0404: "I needed speakers to be able to hear it. I couldn't get the volume - the video at the beginning and a couple of the other videos in it were really helpful, but I couldn't hear it through the computer screen, so I had to go and find some back office somewhere, some speakers to do it."	Cleaned up audio for the intro video
Osteoarthritis	Not enough diversity in videos	0403: "Could have had another example. Just used the same bloke all the way through. Might add variety of someone with OA in a different joint (shoulder/hand etc). Have a	No other videos available – no change. Review in future if resource becomes available

		couple of different scenarios might enable people to reflect further".	to create additional clips.
Osteoarthritis	Practitioners want strategy for managing expectations about X-rays	0401: "I did find the osteoarthritis module interesting, regarding there was a part that said you didn't necessarily need to have an X-ray to diagnose it, and it could be done from just the history. I thought that was quite interesting, but I think a lot of patients probably would want an X-ray to confirm that it wasn't something that, for example, could have a knee replacement, they want to make sure that you're not missing something"	Add a strategy to manage patient expectations about X-Rays.
Osteoarthritis	Nurses value OA learning resources	0401: "as a nurse practitioner we have no separate training in osteoarthritis, and the only thing I might have added into it was an overview page at the beginning about osteoarthritis. So I did then go onto an NHS website just to read a little bit more about it, and I had to do that before I completed the next section."	Sign-posted that there will be more information at the end of the module
Reflections and Goal setting	Practitioners think the reflection and goal setting take too much time.	0401: "I think that's helpful, but realistically we're time-poor, so we might not necessarily do that."	Nothing – this is already brief. Will investigate further in the feasibility trial.
Reflections and Goal setting	Practitioners want a long-term review	0401: "It might be handy, once you've done the training, for a once-yearly, just a quick overview, and here's the video of it all being implemented. Some bullet points might be quite handy."	Added a 2-week email with a reminder to reflect on goals. Consider longer-term review for longer trial (planned feasibility trial too short for yearly review to be relevant)

Reflections and Goal setting	Data is lost if practitioners spend too long on one page.	0404: “The only thing I did find, so I did the reflection bit, and then I obviously managed to hit some button, and it lost it all, so I had to rewrite it.”	Added 'save the page' note to page.

1.5 Supplementary Material 5. Topic Guide for 'Think-aloud interviews'

Primary Care Practitioners' Views about a Digital Training Tool for Communication in Osteoarthritis

Briefing

Thank you for agreeing to take part in a research interview today. I am (*researcher name*), a researcher at University of Southampton. We're interested in your views of our new training tool for primary care practitioners, to enhance their empathic communication skills for patients with osteoarthritis. This is an early version training tool that we are still working on, so there may be parts of it that are not very good yet. We would really value your expert opinion at this early stage. All you have to do is use the training tool as you would normally if I were not here and speak your thoughts out loud whilst you are looking at it. After you have finished looking through the training tool, I would like to have a chat with you about your experiences and your overall views of the materials.

Do you have any questions before we start?

TAKE CONSENT

To help you think aloud you may find it useful to read aloud or tell me what you are clicking on and why.

When you move to a new page, please read aloud the title at the top of the page – this will let the recording know which page you are looking at.

You may find at times I will say aloud what you have clicked on or what page you are looking at – this is just so the recording knows what you are doing on the screen.

This is not a test and you are not being judged. There are no right or wrong answers, so please say any thoughts which spring to mind, even if you think they might not be important. I just want you to say out loud any thoughts which are running through your mind.

Please do feel free to say any negative thoughts you may have about the training tool as these will be really useful in helping us improve it. Your views are really important, the more you can tell us, the better.

This interview is going to be audio recorded. If you would like to stop the interview at any time, for any reason, please let me know and I will stop the recording.

We can take a break at any time – let me know and I will stop the recording. We can either continue after the break, arrange another time to meet or complete the interview where we stopped.

WARM UP TASK

To get you used to speaking aloud as you think, I am going to ask you a question, and I'd like you to tell me what you are thinking as you try to answer it. Try to visualize the place where you live, and think about how many windows there are in that place. As you count up the windows, tell me what you are seeing and thinking about.

START RECORDING

Think-aloud prompts

Remember to state page titles for the recording.

- What are your first impressions of (the information/advice on) this page?
- What are you thinking now?
- Can you tell me more about that? /
- What are your thoughts about that?
- Can you tell me more about what prompted you to click on that?
- What do you think about that advice/using that technique in a consultation?
- What might help you follow that advice?
- What might stop you following that advice?
- How different is that from what you currently do?
- How would that fit in with your consultation?

General questions to follow think-aloud

- Overall, what do you think about training tool and the information given?
- How did you feel about using the training tool?
- What did you particularly like about it?
- Could you tell me about anything you were less keen on?
- What do you think about the overall length of the training tool?
- Can you tell me about anything else that you think might be useful to support communication with patients?
- Which parts of the training did you find most relevant to you? Why these?
- Which of the ideas or techniques are you most likely to try out in a consultation? Why these?
- Which are you least likely to use in a consultation?
- What do you see as the barriers/difficulties to using these ones?
- What would make it easier to use these techniques?
- If you could, what part of the training tool would you change?
- Which parts of the training tool would you not change?
- How would you feel about the training being provided to members of the control group after the trial?

AT THE END OF THE INTERVIEW:

- Ask participant if there is anything else they would like to add
- Advise when stopping the recording
- Thank participant
- Provide opportunity for further questions
- Offer copy of transcript when available
- Offer copy of study findings when available
